# Exploratory Interim Analysis of Serial Plasma and Single-Follicle Follicular Fluid Oxidation–Reduction Potential During Fertility Treatment

**DOI:** 10.3390/biomedicines14102404

**Published:** 2026-10-21

**Authors:** Aris Kaltsas, Stamatis Papaharitou, Kyriakos Papanikolaou, Aristea Sidiropoulou, Katerina Chatzimeletiou

**Affiliations:** 1 Third Department of Urology, Attikon University Hospital, School of Medicine, National and Kapodistrian University of Athens, 12462 Athens, Greece; ariskaltsas@gmail.com; 2 Andrology Laboratory and Sperm Bank, 54622 Thessaloniki, Greece; 3 Fertilia by Genesis, IVF Unit, 55535 Thessaloniki, Greecearisteasidiropoulou@gmail.com (A.S.); 4 Unit for Human Reproduction, First Department of Obstetrics and Gynaecology, Papageorgiou General Hospital, Aristotle University Medical School, 56403 Thessaloniki, Greece; katerinachatzime@hotmail.com

**Keywords:** oxidation–reduction potential, follicular fluid, plasma, assisted reproduction, MiOXSYS, pilot study, ovarian stimulation, oocyte retrieval

## Abstract

**Background/Objectives:** This exploratory interim analysis examined associations between serial plasma oxidation–reduction potential (ORP) and single-follicle follicular-fluid (FF) ORP during fertility treatment. Between-visit measurements and cycle-level outcomes were also described. **Methods:** In 31 cycles from 29 women, plasma was sampled prospectively at cycle day 2 (D2), D6, and pre-trigger, and FF from one follicle at retrieval. Fresh samples were measured in duplicate (MiOXSYS); only means were available. All analyses were post hoc and used Spearman correlations, an intraclass correlation coefficient (ICC), and mixed models with within-family adjustment. **Results:** Plasma–FF correlations were 0.580 at D2 (*n* = 25; 95% confidence interval [CI], 0.178–0.839), 0.715 at D6 (*n* = 23; CI, 0.350–0.924), and 0.866 pre-trigger (*n* = 23; CI, 0.679–0.941). All Holm-adjusted *p* values were ≤0.0027. In 21 women with complete profiles, between-visit correlations were 0.703–0.856 (ICC, 0.788; CI, 0.617–0.872). The mixed-model pre-trigger minus D6 difference was 6.6 mV (CI, 0.6–12.6; Holm-adjusted *p* = 0.0939). No visit contrast or main ORP–outcome association met its adjusted threshold. **Conclusions:** Plasma and FF ORP were associated in this small interim cohort. Neither biological stability nor a true temporal change was established, and assay precision, agreement, and prognostic value remain unknown. Clinical application is not supported.

## 1. Introduction

Follicular fluid (FF) surrounds the developing oocyte and reflects its biochemical environment. Several redox markers in FF have been examined in relation to fertilization and embryo development, and the results differ according to the marker and the outcome [1]. In one study of women undergoing assisted reproduction, lower FF glutathione and higher 8-hydroxy-2′-deoxyguanosine were associated with lower fertilization rates after intracytoplasmic sperm injection (ICSI). In the same study, total antioxidant capacity (TAC) did not differ significantly between groups defined by the embryological outcomes. Antioxidant measures and markers of oxidative damage should therefore be considered separately [2].

Oxidation–reduction potential (ORP) is an electrochemical measure of the redox state of a sample. Static ORP is expressed in millivolts and has been measured in FF with the MiOXSYS system. In a study of 341 ICSI cycles, FF ORP had a weak inverse association with the fertilization rate, whereas its associations with oocyte maturation and blastocyst formation rates were not statistically significant. FF ORP therefore merits further study, but its ability to predict reproductive outcomes has not been established [3].

It is less clear how ORP in the circulation relates to ORP in the follicle. FF is formed from plasma exudate and from the secretory products of granulosa and theca cells [1], so some relationship between the two measurements is expected. Plasma has the practical advantage that it can be sampled at several monitoring visits before retrieval. In heparinized plasma, static ORP responded to added oxidant and reductant, and fresh samples were the most suitable for measurement [4]. In 20 adults, repeated fasting samples gave consistent within-person values over several weeks [5]. Direct comparisons of plasma and FF are limited to two conference abstracts, both with samples taken on the day of oocyte retrieval. One reported positive correlations in oocyte donors and in patients, and the other found no significant association between plasma and FF static ORP [6,7]. The relationship between plasma ORP earlier in the treatment cycle and FF ORP at retrieval is not known.

The time of sampling may also matter. In women with endometriosis undergoing ICSI, serial measurements showed different temporal patterns for circulating glutathione and TAC [8]. These are biochemical markers and not ORP, but the observation supports measurement at defined treatment visits.

The approved protocol concerned ORP in blood and FF as a prognostic marker of ovarian stimulation and treatment-cycle outcomes. This exploratory interim report describes those outcome associations and two additional questions: the association of plasma ORP at three treatment visits with single-follicle FF ORP at retrieval, and the association of plasma ORP between visits. These two questions were not among the stated protocol aims. All analyses were specified after data collection and are hypothesis-generating.

## 2. Materials and Methods

### 2.1. Study Design and Participants

This exploratory interim analysis uses the initial available cohort of an ongoing prospective observational pilot study of consenting women undergoing fertility treatment. Samples for this interim cohort were collected at the Fertilia by Genesis IVF Unit, Thessaloniki, Greece, between February and September 2026, after ethics approval on 20 January 2026. Oocyte retrieval, when performed, took place at the same unit. Exact specimen-collection timestamps were unavailable, and intervals between visits were not analyzed. Protocol eligibility criteria were age below 40 years, body mass index (BMI) below 30 kg/m^2^, and anti-Müllerian hormone (AMH) concentration of 0.7–5.0 ng/mL. AMH-based eligibility was assessed during clinical screening. Numerical screening AMH values for two women were not transferred to the analysis dataset; their observations were retained in analyses not requiring AMH. Known polycystic ovary syndrome (PCOS) and endometriosis were exclusion criteria, whereas smoking was not. Treatment comprised antagonist and long protocols, natural and modified natural cycles, and cycles for oocyte cryopreservation. A woman could contribute more than one cycle. Screening and refusal counts were unavailable.

The approved protocol was entitled “The oxidation–reduction potential (ORP) in blood and follicular fluid as a prognostic marker of the outcome of ovarian stimulation and of the in vitro fertilization cycle”. The participant information described two aims: associations of blood and FF ORP with oocyte quality and fertilization, and associations with the hormonal profile, demographic characteristics, lifestyle, and ovarian stimulation. The 31 available cycles form the initial exploratory cohort. No formal sample-size calculation or hierarchy of confirmatory endpoints was prespecified. The study was not registered, and no fixed final recruitment target was prespecified. A prespecified interim stopping rule was not documented in the study materials available for this report. The present sample size reflects the available records and is not a power-based target.

### 2.2. Clinical Monitoring and Sample Collection

Blood was sampled at three visits. The first was on cycle day 2 (D2), before the start of stimulation where applicable. The second was the D6 monitoring visit, which was stimulation day 6 in stimulated cycles and menstrual-cycle day 6 in natural cycles. The third was before the trigger for final oocyte maturation. The trigger was choriogonadotropin alfa (Ovitrelle; Merck Europe B.V., Amsterdam, The Netherlands), given approximately 36 h before retrieval. One third-visit sample came from a natural cycle without a trigger and was excluded from the pre-trigger analyses.

FF was collected at oocyte retrieval from the follicle with the largest ultrasound-measured diameter, designated as the dominant follicle, and was not pooled. Individual follicle diameters, aspiration order, use of follicular flushing, and anesthetic agent were not available in the study records. Follicles were not matched to individual oocytes or embryos; FF ORP was related only descriptively to outcomes of the whole cycle.

### 2.3. Ethical Approval

The research protocol and the participant information and consent documents were approved by the Bioethics and Ethics Committee of the Medical School, Aristotle University of Thessaloniki. The reference number was 89/2026 (committee meeting, 13 January 2026; approval letter, 20 January 2026). All participants gave written informed consent, which covered the repeated treatment cycles included in the study. All samples included in this analysis were collected after approval, through September 2026.

### 2.4. Sample Processing and ORP Measurement

Peripheral blood was collected into lithium-heparin tubes, left at room temperature for 30 min, and centrifuged at 400× *g* for 30 min (Rotofix 32; Hettich, Tuttlingen, Germany). Plasma ORP was measured in fresh samples that had not been frozen. The use of heparin and of fresh samples follows published optimization data for plasma ORP [4].

The procedure accepted clear FF aspirates without visible blood and excluded visibly blood-contaminated aspirates, including light-pink ones. Accepted samples were passed through a syringe filter and analyzed fresh, without centrifugation. The recorded filter pore-size range was 1–5 μm; the exact pore size, membrane material, and manufacturer were unavailable. Visual assessment did not provide an objective measure of blood contamination.

Static ORP was measured with the MiOXSYS^®^ analyzer and disposable sensors (UAB Caerus Biotechnologies, Vilnius, Lithuania). A sensor was inserted into the analyzer, and 30 μL of sample was applied to the sample port. Routine analyzer checks according to the manufacturer’s instructions were performed, but quantitative quality-control records were unavailable. Samples were measured in duplicate on separate sensors, immediately after exposure to air, with a target interval of 2 min. Individual exposure and measurement timestamps were unavailable, so compliance with this window could not be verified sample by sample. Only the recorded arithmetic mean of the two readings, in millivolts (mV) without normalization, was available for analysis. Individual readings, sensor-lot data, and operator records could not be recovered; within-sample repeatability, between-run precision, and drift could therefore not be assessed. ORP was measured before fertilization and blastocyst development were assessed. The Supplementary Methods identify further undocumented procedural details.

### 2.5. Embryological Outcomes

Outcomes were recorded per treatment cycle as retrieved-oocyte, metaphase II (MII) oocyte, normally fertilized two-pronuclear (2PN) oocyte, and day-5 and day-6 blastocyst counts. The oocyte maturation proportion was the MII count divided by retrieved oocytes. Additional clinical records identified ICSI and total injected-oocyte counts in 20 of 21 retrievals not limited to oocyte cryopreservation; these fields were missing for one cycle. The original exploratory correlations used the 2PN/recorded-MII proportion in 16 cycles with both counts available. Confirmed injected counts equaled recorded MII counts in these 16 cycles. The 2PN/total-injected proportion for all 20 documented ICSI cycles is reported descriptively in Table S10, without additional correlation tests; total injected counts are not assumed to establish maturity at injection. The recorded blastocyst/2PN proportion was the blastocyst count divided by the 2PN count; extended-culture eligibility and transfers or cryopreservation before day 5 were unverified, and no morphology grade was applied. Numerical antral follicle count (AFC) was available for 25 retrieval cycles. Treatment indication remained unavailable. These summaries do not establish laboratory performance or prognostic value.

In one cycle, 10 retrieved oocytes and 7 germinal-vesicle oocytes were recorded, but the MII count was missing. A sensitivity analysis assumed that the remaining three oocytes were MII, and this assumption was not used in the main analyses. Rates were calculated only when the numerator was available and the denominator was positive. Missing outcomes were not imputed, and confirmed blastocyst counts of zero were retained. A post hoc sensitivity analysis coded documented cancellations for poor response as zero retrieved oocytes and used the first recorded cycle of each woman.

### 2.6. Statistical Analysis

All analyses were specified after data collection and are exploratory. Effect estimates and their uncertainty are emphasized. The authors selected the test families after data collection; adjustment within a family does not control multiplicity across the full analysis set or convert a post hoc result into confirmatory evidence. Supplementary Table S1 lists each family, the stated aim addressed, and the timing of its addition, including the age analyses expanded during this revision.

Descriptive data are given as mean ± standard deviation (SD) and median (interquartile range [IQR]). Baseline characteristics were summarized once per woman, and ORP measurements and outcomes per cycle. Each correlation included at most one observation per woman. Only one of the three cycles contributed by one woman reached retrieval and had a complete plasma profile, so the plasma–FF, complete-profile, and outcome correlations required no choice between her cycles. Her first recorded D2 value was used for baseline participant-characteristic analyses. For age associations, the first available ORP measurement at each sampling stage was selected per woman before pairwise analysis, by recorded cycle order and without regard to value or treatment outcome.

Associations between plasma and FF ORP were described with Spearman correlations, with Holm adjustment across the three visits. Between-visit rank associations were described in women with complete three-visit profiles using Spearman coefficients. A two-way mixed-effects, absolute-agreement, single-measurement intraclass correlation coefficient (ICC) was also calculated. This ICC summarizes the observed between-visit agreement, which combines biological and analytical variation; it does not estimate assay repeatability or establish biological stability of absolute ORP.

Change within the cycle was estimated with a linear mixed model of all available plasma measurements, with visit as a fixed effect and a random intercept for woman. Holm adjustment was applied to the three visit contrasts. Models were fitted by restricted maximum likelihood. A model with cycle nested within woman was fitted as a sensitivity analysis. Only one woman contributed repeated cycles, which limits the estimation of this additional variance component. The first analysis had been a complete-case comparison in women with all three visits, with the Friedman test and paired Wilcoxon signed-rank tests. Because the mixed model uses all measurements, the complete-case comparison is reported as a sensitivity analysis.

Associations of ORP with outcomes were described with Spearman correlations. Benjamini–Hochberg adjustment was applied separately to five FF–outcome associations, 15 plasma–outcome associations, and 12 associations of plasma ORP changes or profile features with oocyte and blastocyst counts. The profile features were the minimum, maximum, and range of the three plasma values. Four additional correlations related third-visit estradiol and progesterone to pre-trigger plasma ORP and FF ORP without significance testing. Age was related to D2, D6, and pre-trigger plasma ORP and FF ORP as a separate post hoc family of four correlations with Holm adjustment (Supplementary Table S9). The untriggered natural-cycle specimen was excluded from pre-trigger analyses.

Correlation and Friedman *p* values used 99,999 permutations, and paired Wilcoxon tests used sign permutations. Before rank-based analyses, differences were rounded to source-measurement precision to preserve ties. Pointwise 95% confidence intervals (CIs) used 20,000 bootstrap resamples of women or paired observations; mixed-model CIs are Wald intervals. CIs were not adjusted for multiplicity. Sensitivity analyses addressed missing visits, protocol type, the unconfirmed MII count, and one D2 specimen annotated as low volume/hemolysis. Tests were two-sided; *p* < 0.05 after within-family adjustment was used as a descriptive threshold. Unadjusted *p* values and all post hoc analyses were interpreted descriptively. No confounder-adjusted association or prediction model was fitted, and no clinical cutoff was estimated. Software and resampling details are given in the Supplementary Methods.

## 3. Results

### 3.1. Study Population and Sample Availability

**Figure 1:**
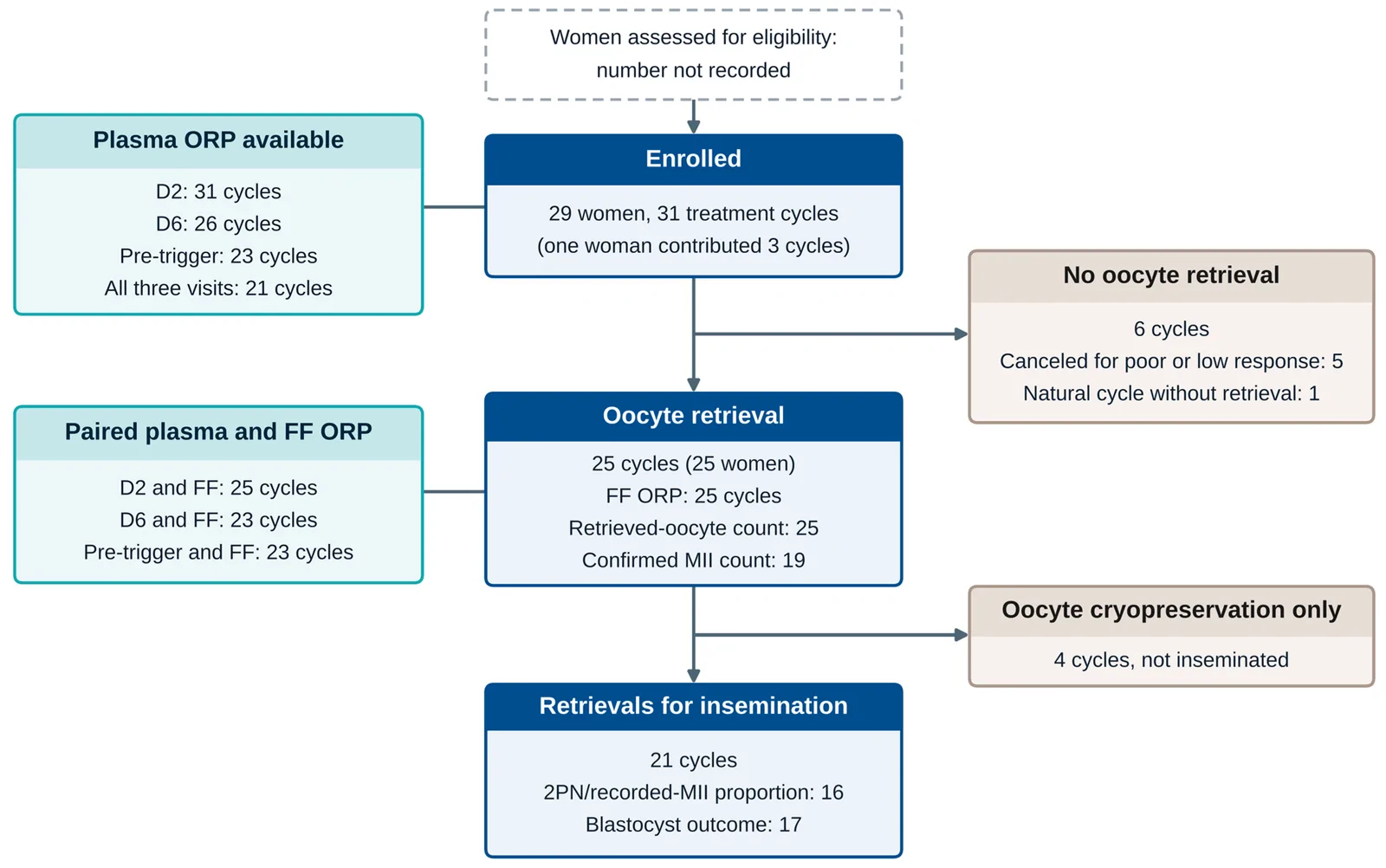
Flow of women and treatment cycles. The boxes give the number of cycles with plasma ORP at each visit, with oocyte retrieval and follicular-fluid (FF) ORP, and with evaluable outcomes. The number of women assessed for eligibility was not recorded. One third-visit specimen from an untriggered natural cycle was not counted as pre-trigger. Retrievals for insemination are those not limited to oocyte cryopreservation. ORP, oxidation–reduction potential; D2, cycle day 2; D6, stimulation day 6 (menstrual-cycle day 6 in natural cycles); MII, metaphase II; 2PN, two pronuclei.

The analysis included 31 treatment cycles from 29 women, one of whom contributed three cycles. Figure 1 shows the flow of women and cycles through sampling, retrieval, and outcome assessment.

Mean age was 34.1 ± 3.7 years. Numerical screening AMH values were missing from the analysis dataset for two women. There were 24 antagonist cycles, three long-protocol cycles, three natural cycles, and one modified natural cycle. Twenty-five cycles reached retrieval, five were canceled because of poor or low ovarian response, and in one natural cycle no retrieval was performed. Four retrievals were for oocyte cryopreservation only. Eight women were smokers and 20 were non-smokers, and smoking status was missing for one woman. Numerical AFC was available for the 25 retrieval cycles from 25 women: median 12 (IQR, 10–13; range, 1–20). Table 1 summarizes participant characteristics, ORP measurements, and outcome counts; Table S10 gives the additional clinical descriptors, and Table S2 gives CIs for median ORP values.

Complete three-visit plasma profiles were available in 21 of 31 cycles (67.7%), and FF ORP in all 25 retrievals. Confirmed MII counts were available in 19 of 25 retrievals, and blastocyst outcomes in 17 of the 21 retrievals that were not limited to cryopreservation. Supplementary Table S3 gives the reasons for cancellation and for missing outcomes.

### 3.2. Plasma ORP and Follicular-Fluid ORP

**Figure 2:**
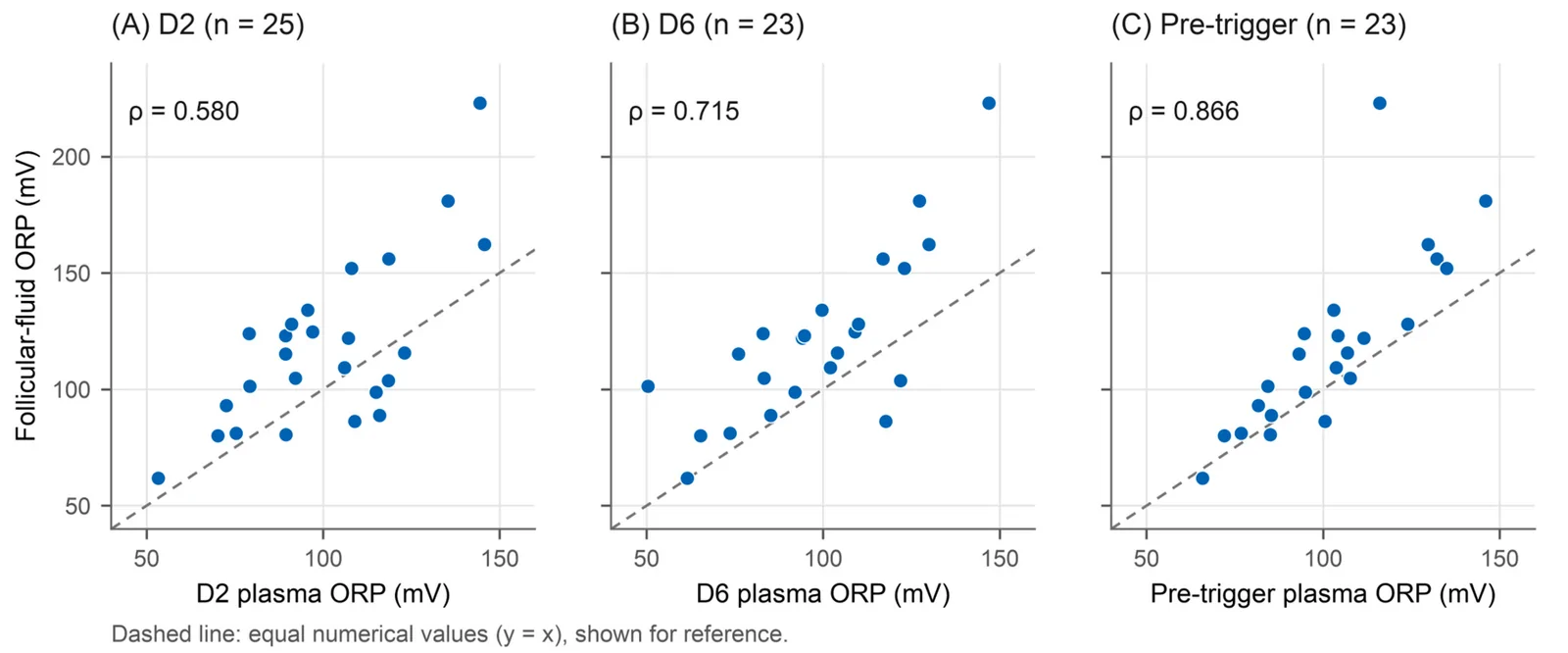
Follicular-fluid (FF) ORP at retrieval plotted against plasma ORP at (**A**) D2, (**B**) D6, and (**C**) pre-trigger. Each point represents one cycle from one woman. ρ is the Spearman coefficient, and the dashed line indicates numerically equal values. Plasma and FF were collected at different times and from different matrices, so the equality line does not indicate analytical agreement. ORP, oxidation–reduction potential; D2, cycle day 2; D6, stimulation day 6 (menstrual-cycle day 6 in natural cycles).

Plasma ORP at D2, D6, and pre-trigger was positively associated with FF ORP at retrieval in the same cycle (Table 2). The coefficients were 0.580, 0.715, and 0.866, with pointwise 95% CIs of 0.178–0.839, 0.350–0.924, and 0.679–0.941, respectively; all Holm-adjusted *p* values were ≤0.0027. These are exploratory associations. The coefficients were not formally compared between visits. Figure 2 shows the paired measurements.

In the 21 women with all three plasma measurements and FF, the coefficients were 0.596, 0.758, and 0.848. When the analysis was restricted to antagonist cycles, they were 0.661, 0.835, and 0.900. The pre-trigger coefficient was 0.864 after omission of the highest FF value (223.0 mV; *n* = 22), and the D2 coefficient was 0.533 after omission of the hemolysis-flagged specimen (*n* = 24). These sensitivity analyses are reported in Supplementary Table S4.

FF ORP was higher than pre-trigger plasma ORP in 19 of 23 cycles, with a median paired difference of 10.4 mV (95% CI, 4.3–18.8; unadjusted *p* = 0.0002). The median differences from D2 and D6 plasma ORP were 16.5 and 21.5 mV (Supplementary Table S4).

### 3.3. Plasma ORP Between Visits

**Figure 3:**
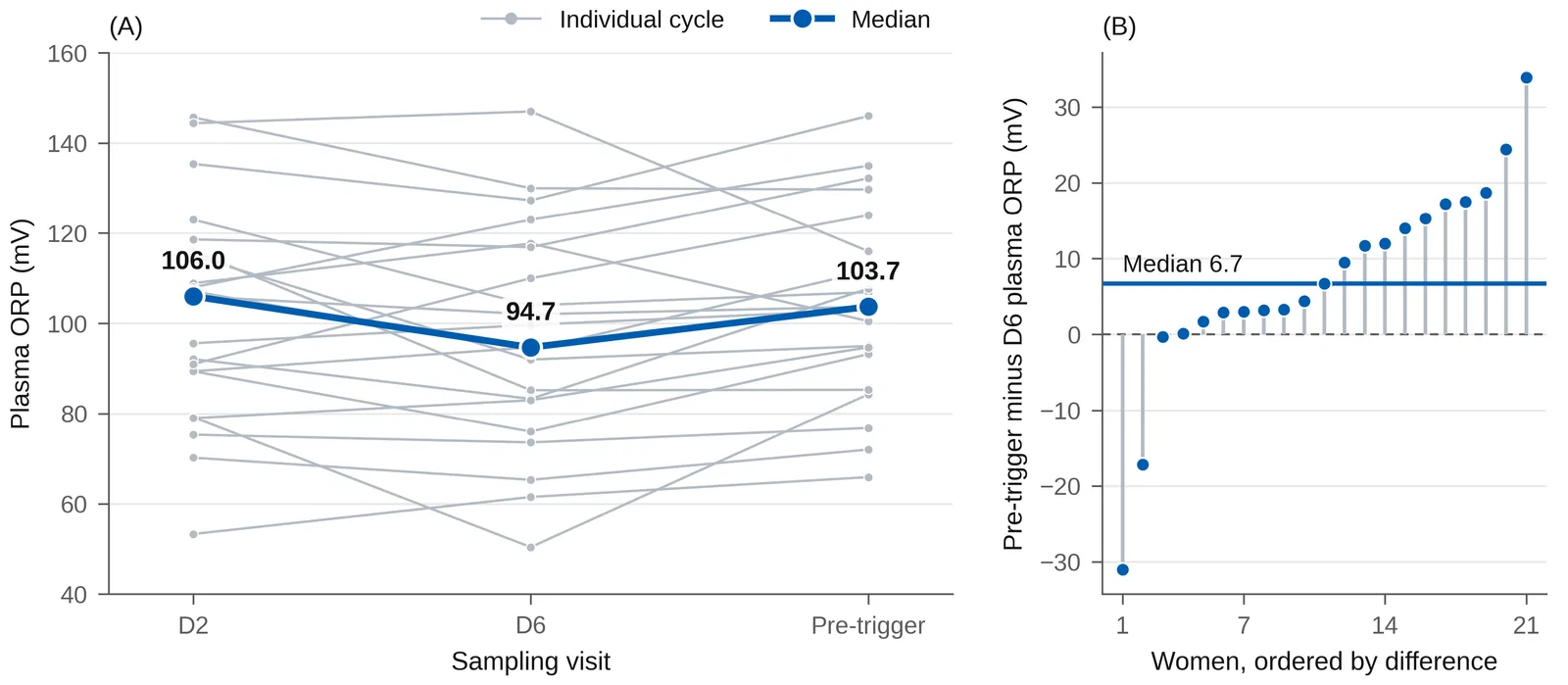
Plasma ORP in the complete three-visit cohort (21 cycles from 21 women). (**A**) Measurements at the three sampling visits. Gray lines connect the measurements of one cycle, and the blue line and the values show the median at each visit. Visits are shown as categories, and horizontal spacing does not represent elapsed time. (**B**) Paired differences between the pre-trigger and D6 visits, ordered by size; the blue line marks the median. ORP, oxidation–reduction potential; D2, cycle day 2; D6, stimulation day 6 (menstrual-cycle day 6 in natural cycles).

Recorded plasma ORP values were positively correlated between visits. In the 21 women with complete profiles, Spearman coefficients were 0.829 between D2 and D6, 0.856 between D6 and pre-trigger, and 0.703 between D2 and pre-trigger (Table 3). The ICC was 0.788 (95% CI, 0.617–0.872). These summaries describe the observed measurements and cannot distinguish persistent biological differences from persistent analytical or handling effects. Figure 3 shows the individual profiles.

In the mixed model of all 80 plasma measurements from 29 women, the estimated pre-trigger minus D6 difference in recorded ORP was 6.6 mV (95% CI, 0.6–12.6; unadjusted *p* = 0.0313; Holm-adjusted *p* = 0.0939). No visit contrast met the family-adjusted threshold. In antagonist cycles (62 measurements from 22 women), the corresponding estimate was 6.9 mV (95% CI, −0.3 to 14.0). The nested-cycle model gave 6.7 mV for all cycles (95% CI, 0.9–12.5; Holm-adjusted *p* = 0.0672). These estimates cannot be separated from measurement noise or drift.

In the complete-case analysis of 21 women, median plasma ORP was 106.0 mV at D2, 94.7 mV at D6, and 103.7 mV at pre-trigger (Friedman permutation *p* = 0.0045). The median difference between pre-trigger and D6 was 6.7 mV (95% CI, 3.0–14.0; Holm-adjusted *p* = 0.0149), and neither contrast with D2 was significant. Median D2 ORP was lower in the 10 cycles outside the complete cohort (87.0 mV) than in the complete cohort (106.0 mV). Supplementary Table S5 gives the complete-case, sensitivity, and additional mixed-model results.

### 3.4. ORP and Participant Characteristics

Median D2 plasma ORP was 112.8 mV (IQR, 91.4–126.1) in 8 smokers and 96.3 mV (IQR, 77.4–108.2) in 20 non-smokers (unadjusted *p* = 0.1270). Median FF ORP was 118.8 mV in 8 smokers and 109.3 mV in 17 non-smokers (unadjusted *p* = 0.4747). D2 plasma ORP and FF ORP showed small correlations with BMI and AMH (|ρ| ≤ 0.197; all unadjusted *p* ≥ 0.34). These unadjusted comparisons do not exclude confounding.

Correlations of age with ORP were close to zero, with wide intervals. The Spearman coefficients were 0.026 for D2 plasma ORP (*n* = 29; pointwise 95% CI, −0.427 to 0.426) and 0.110 for D6 plasma ORP (*n* = 25; CI, −0.347 to 0.523). For pre-trigger plasma ORP and FF ORP, they were 0.036 (*n* = 23; CI, −0.365 to 0.423) and 0.146 (*n* = 25; CI, −0.282 to 0.535). All Holm-adjusted *p* values were 1.0000 (Supplementary Table S9). These intervals and the restricted age range prevent a conclusion that ORP is independent of age.

Third-visit hormone values were available for 19 retrieval cycles. Median estradiol was 1800.0 pg/mL and median progesterone 0.98 ng/mL. Estradiol correlations were −0.104 with pre-trigger plasma ORP and −0.279 with FF ORP; progesterone correlations were −0.335 and −0.485. The progesterone–FF pointwise CI was −0.733 to −0.112 (Supplementary Table S6). These four post hoc, unadjusted associations do not establish an independent hormonal relationship. The recorded progesterone maximum of 6.40 ng/mL was rechecked and confirmed as the clinical laboratory result. Precise sampling timestamps were unavailable in the analysis records.

### 3.5. ORP and Embryological Outcomes

Table 4 describes MII counts and the recorded embryological proportions. The original 2PN/recorded-MII correlation analyses used 16 of the 21 retrievals not limited to cryopreservation. Additional clinical records documented ICSI and total injected counts in 20 of these 21 cycles. Their median recorded 2PN/total-injected proportion was 81.7% (IQR, 66.7–86.5%; range, 30.0–100.0%; Table S10). In the 16 cycles with recorded MII, the confirmed injected counts matched MII counts, so the original ratio values and correlations were retained. No ORP correlations were calculated for the expanded 20-cycle injected-count cohort. All outcome associations remain descriptive, and extended-culture denominators remain unverified.

The five FF–outcome coefficients ranged from −0.393 to 0.230, all pointwise 95% CIs included zero, and no association met the Benjamini–Hochberg-adjusted threshold (adjusted *p* = 0.3318–0.6447; Table 5). Their wide intervals are compatible with associations in either direction and do not establish absence of a relationship.

The 15 plasma–outcome associations were not significant after adjustment either. The same applied to the 12 associations of plasma ORP changes and profile features with oocyte and blastocyst counts (Supplementary Table S7). The coefficients for plasma ORP and oocyte count were positive at all three visits (0.357, 0.379, and 0.342). When the unconfirmed MII value was retained, the FF coefficients became −0.184 for maturation (*n* = 20) and −0.480 for the 2PN/recorded-MII proportion (*n* = 17) (Supplementary Table S8).

In the post hoc sensitivity analysis that coded poor-response cancellations as zero retrieved oocytes, the correlation of D2 plasma ORP with oocyte count was 0.404 (*n* = 28; unadjusted 95% CI, 0.019–0.685). The D6 correlation was 0.424 (*n* = 23), and its CI included zero. Among first cycles, median D2 plasma ORP was 101.5 mV in those that reached retrieval and 93.2 mV in those canceled for poor response (Supplementary Table S3). These estimates are hypothesis-generating only.

## 4. Discussion

### 4.1. Principal Findings

In this exploratory interim cohort, recorded plasma ORP at D2, D6, and pre-trigger was associated with single-follicle FF ORP at retrieval. Plasma measurements were also correlated between visits. A shared component is compatible with FF composition and an earlier retrieval-day report using the same analyzer [6]. The serial observations extend the descriptive evidence to plasma sampled before retrieval, but they cannot identify the biological or analytical source of the association. The outcome analyses addressing the original prognostic purpose were inconclusive and did not establish prognostic performance.

### 4.2. Plasma–FF Associations and Between-Visit Rank Correlations

Plasma and FF have been compared directly only in the two conference abstracts. The 2023 report included 15 donors and 55 patients and found retrieval-day correlations of 0.90 and 0.51 [6]. The 2026 report covered 321 ICSI cycles, with paired samples in 50 women, and found no significant static-ORP correlation in pooled FF [7]. In the 2023 report, the difference between FF and plasma was in the direction opposite to the present one. Mean FF ORP was lower than plasma ORP in patients (136.2 vs. 148.3 mV) and in donors (160.3 vs. 170.3 mV) [6]. Here, FF ORP was higher than pre-trigger plasma ORP in 19 of 23 cycles. Median pre-trigger plasma ORP was also lower here (103.0 mV) than the patient mean in that report. The discrepancy may be due to differences in sampling, processing, or patient populations, and the available data cannot separate these explanations. Possible methodological differences include the time of plasma sampling, FF filtration, the anticoagulant, and the time to measurement. Values from different studies should therefore be compared with caution.

Absolute values also differed from other reports. The 341-cycle study, which also excluded blood-contaminated FF, reported a median FF ORP of 82.49 mV [3], compared with 115.2 mV here. Median plasma ORP in the complete cohort (94.7–106.0 mV) was lower than the values of approximately 128–136 mV reported for healthy adults with a related analyzer [4,5]. None of these values can be used as a reference level by another laboratory.

The plasma correlations between visits (0.703–0.856) were similar in size to the pre-trigger plasma–FF correlation in the same 21 women (0.848). These rank associations are compatible with persistent differences between women, but do not establish stable absolute biological values. Repeated plasma sampling in 20 adults also found a persistent component [5], although that study cannot validate the present procedure. Stable handling effects, unmeasured sensor factors, or biological characteristics could contribute to the observed ranking. Plasma ascorbate is one candidate for future study: associations with static ORP and an in vitro ORP response have been reported [4,5,9]. Diet and supplement use were unavailable here.

A shared systemic contribution is plausible, but the data cannot partition systemic and follicular sources. Becatti et al. measured plasma and FF oxidative-stress biomarkers and a derived redox index in women undergoing assisted reproduction [10]; their findings provide context for studying both compartments, but did not validate ORP interchangeability. Here, ρ^2^ ≈ 0.75 for pre-trigger plasma and FF concerns ranked measurements. It cannot quantify the proportion of biological follicular redox variation attributable to plasma, demonstrate causality, or establish agreement or predictive value.

### 4.3. Within-Cycle Change

The mixed model estimated a pre-trigger minus D6 difference of 6.6 mV, with Holm-adjusted *p* = 0.0939; the nested-cycle variant gave *p* = 0.0672. The complete-case analysis estimated a median paired difference of 6.7 mV with adjusted *p* = 0.0149. The estimates are similar in direction and size, but the inferences differ because the analyses use different populations and estimands. The complete-case analysis used paired rank-based comparisons in 21 women, and the mixed model a mean visit contrast from 80 measurements in 29 women. They also use different uncertainty calculations. Incomplete cycles had lower D2 ORP, and the mixed model’s missing-at-random assumption may fail if missingness relates to ovarian response. The all-available mixed model was retained as the principal temporal analysis, and the complete-case analysis as sensitivity evidence. Neither establishes a biological change, because precision and drift are unquantified. Published repeatability estimates from related platforms and different preparations [4,5,9] cannot supply the missing precision of this procedure. Other circulating redox markers have shown visit-dependent values [8,11], but that does not resolve the analytical uncertainty here.

### 4.4. Outcome Associations

The outcome analyses address the original prognostic aim, but the evaluable samples are small and the CIs wide. Failure to meet a multiplicity-adjusted threshold does not establish absence of an association. Positive coefficients for plasma ORP and oocyte count, including the cancellation sensitivity analysis, are exploratory patterns that cannot establish a useful marker. Restriction of the main analyses to cycles reaching retrieval can introduce selection; first cycles canceled for poor response had lower recorded D2 ORP. Moreover, one unmatched follicle, selected outcome-analysis subsets, and unverified extended-culture denominators cannot support follicle-specific prognosis or laboratory performance claims.

The 341-cycle study reported a weak association between FF ORP and fertilization (ρ = −0.126) [3]. Findings in diminished ovarian reserve [12], in a separate report of 212 cycles [13], and in conference abstracts [7,14] were not consistent across endpoints. The three full reports come from one center, share an ethics identifier, and have overlapping recruitment periods [3,12,13]. Participants may therefore overlap, and the reports should not automatically be regarded as independent replications.

### 4.5. Measurement Scope

Static ORP is an electrochemical potential under the conditions of measurement. It does not directly quantify reactive oxygen species, cellular damage, or antioxidant reserve. ORP differs between assisted reproduction media [15], so the same analyzer and unit do not provide a common cutoff for plasma and FF. Thresholds for semen ORP cannot be transferred either. Normalized ORP discriminated abnormal semen parameters in one multicenter study, whereas in repeated samples several associations were driven by the normalization to sperm concentration [16,17]. For plasma on a related analyzer, reported CVs were below 2% in a small repeatability experiment [4], 4.6% in fresh triplicates [9], and 5.3% across three days in pooled plasma supplemented with α-tocopherol [5]. These figures do not apply to the present procedure, in which the analyzer and the plasma preparation were different. Follicle-level studies that match each follicle to its oocyte address a different question and must allow for clustering within women [18,19].

### 4.6. Strengths and Limitations

The strengths of the study are sampling at defined treatment visits, explicit denominators for every analysis, the use of one cycle per woman in each correlation, and multiplicity adjustment within families.

The main limitation is analytical. Individual duplicate readings, quantitative quality-control results, sensor lots, and operator records were unavailable. Within-sample repeatability, drift, and the analytical contribution to between-visit variation are therefore unknown. Sample pH, measurement temperature, and collection-to-measurement intervals were not recorded. The 2 min air-exposure target lacks individual timestamps and cannot be verified retrospectively.

The 30 min interval before plasma separation and centrifugation at 400× *g* did not establish a platelet-poor preparation. Residual platelets or other cells, unrecorded pH and temperature, and handling time could affect the measured signal independently of follicular biology. In FF, visual screening cannot quantify blood contamination, and filtration without a validated comparison leaves its influence unresolved. Plasma optimization work shows that sample preparation can alter ORP [4], but it does not quantify the effects of this study’s specific plasma or FF procedure. The magnitude and direction of these potential biases cannot be reconstructed from recorded means.

The cohort was small and heterogeneous, combining four protocol types with different endocrine states at nominally similar visits. Antagonist-only analyses cannot exclude protocol confounding, and the descriptive smoking comparisons cannot rule out smoking-related bias; supplement exposure was unavailable. Cycles with missing visits had lower D2 ORP, screening counts were unknown, and cancellations may violate the mixed-model missing-at-random assumption. A single follicle was not linked to its oocyte or embryo. ICSI and injected counts were documented for 20 of 21 non-cryopreservation retrievals, and numerical AFC for the 25 retrieval cycles only. Treatment indication and extended-culture eligibility remained unavailable. Original 2PN/MII correlations used 16 cycles with recorded MII and were not extended to the four additional injected-count records. Recorded outcome proportions remain descriptive. AMH-based screening was confirmed, but the numerical screening results for two women were unavailable for independent rechecking in the analysis records. Incomplete calendar records prevented reconstruction of intervals between visits. Pregnancy linkage was unreliable. The restricted age range of 27–39 years, BMI/AMH criteria, and exclusion of PCOS and endometriosis limit generalizability. Wide age-association intervals do not demonstrate independence from age.

All analyses were specified after data collection. Including the expanded age analysis, 109 estimates in 18 author-selected families were derived from 31 cycles (Supplementary Table S1). Adjustment applies only within families; CIs are pointwise, confounder adjustment was not performed, and bootstrap coverage may be imperfect at these sample sizes. The analysis therefore supports hypothesis generation rather than confirmation.

### 4.7. Future Research

The method should be validated first. Validation requires retained replicate readings in fresh lithium-heparin plasma and in FF, between-run and between-day experiments, and records of pH, temperature, timing, sensor lot, and operator. Plasma should be prepared by a validated platelet-poor method. For FF, an objective measure of blood contamination and a comparison of filtered and unfiltered aliquots are needed. Fresh and thawed plasma should not be mixed, because one freeze–thaw cycle lowered ORP in heparin plasma by 6 mV [4]. Sampling of plasma and FF at the same time at retrieval, a predefined follicle, and linkage to the embryo would separate matrix effects from timing. Measurement of plasma ascorbate, with structured records of diet and supplement use, would test one possible source of the persistent difference between women.

A clinical study is justified only if the assay proves reproducible. It would ask whether plasma ORP adds prognostic information to age, AMH, and antral follicle count. Such a study should be powered for the incremental discrimination or calibration of a prespecified model and should include independent validation. For orientation only, an approximate calculation for a single correlation of 0.30 or 0.20, with two-sided α = 0.05 and 80% power, gives 89 or 198 independent women, respectively. The formula and its limitations are given in the Supplementary Methods [20].

## 5. Conclusions

This exploratory interim analysis found positive associations between recorded plasma ORP at three treatment visits and single-follicle FF ORP at retrieval. Plasma measurements were also correlated between visits, but these findings do not establish biological stability, assay agreement, or prognostic value. The temporal difference cannot be distinguished from measurement noise or drift, and cycle-level outcome associations remain inconclusive. Retained replicate measurements and validated plasma and FF handling are required before a larger, prospectively planned study can test whether plasma ORP adds prognostic information beyond established clinical factors. The present data do not support a clinical cutoff, prediction tool, or treatment indication.

## Figures and Tables

**Table 1:** Participant characteristics, ORP measurements, and oocyte and blastocyst counts.

Measure	*n* Records/Women	Mean ± SD	Median (IQR)	Range
Age (years)	29/29	34.1 ± 3.7	34.0 (32.0–37.0)	27.0–39.0
BMI (kg/m^2^)	29/29	23.7 ± 3.1	22.7 (22.0–27.0)	18.0–29.4
AMH (ng/mL)	27/27	1.80 ± 0.89	1.60 (1.05–2.60)	0.70–3.65
D2 plasma ORP (mV)	31/29	97.7 ± 22.9	95.6 (81.0–112.0)	53.3–145.7
D6 plasma ORP (mV)	26/25	95.6 ± 24.4	94.4 (77.0–115.2)	50.4–147.0
Pre-trigger plasma ORP (mV)	23/23	102.4 ± 21.2	103.0 (85.2–113.8)	65.9–146.0
Follicular-fluid ORP (mV)	25/25	118.0 ± 35.9	115.2 (93.0–128.0)	61.8–223.0
Retrieved oocytes (count)	25/25	8.0 ± 3.7	8.0 (7.0–10.0)	1.0–15.0
Total blastocysts (count)	17/17	3.2 ± 2.6	3.0 (1.0–4.0)	0.0–10.0
Total gonadotropin dose (IU) *	21/21	3213.1 ± 624.0	3300.0 (3000.0–3600.0)	1350.0–4550.0

Baseline characteristics were summarized once per woman. ORP and outcome rows use the available cycle-level observations; the *n* column gives observations and distinct women. Pre-trigger measurements exclude the untriggered natural-cycle specimen. Blastocyst counts include culture days 5 and 6 and exclude cryopreservation-only cycles. * Dose is summarized for the 21 stimulated retrieval cycles with totals recorded in IU; two natural cycles had no dose entry, and two entries with formulation-specific units were excluded. SD, standard deviation; IQR, interquartile range; BMI, body mass index; AMH, anti-Müllerian hormone; D2, cycle day 2; D6, stimulation day 6 (menstrual-cycle day 6 in natural cycles); ORP, oxidation–reduction potential; IU, international units.

**Table 2:** Associations between plasma ORP and follicular-fluid ORP from the same treatment cycle.

Plasma Sampling Visit	Paired Cycles/ Women, *n*	Spearman ρ	95% CI for ρ	Holm-Adjusted *p*
D2	25/25	0.580	0.178–0.839	0.0027
D6	23/23	0.715	0.350–0.924	0.0004
Pre-trigger	23/23	0.866	0.679–0.941	<0.0001

Each analysis used paired plasma and FF measurements from the same cycle, with one cycle per woman. The D6 and pre-trigger analyses each included 23 women, but not the same women. *p* values are two-sided permutation estimates (99,999 permutations) with Holm adjustment across the three correlations. CIs are pointwise percentile bootstrap intervals (20,000 resamples of paired observations). ORP, oxidation–reduction potential; FF, follicular fluid; CI, confidence interval.

**Table 3:** Plasma ORP between visits: rank correlations and mixed-model contrasts.

Visit Pair	Spearman ρ (95% CI)	Later Minus Earlier Visit, mV (95% CI)	*p*	Holm-Adjusted *p*
D2 and D6	0.829 (0.549–0.943)	−4.6 (−10.1 to 1.0)	0.1053	0.2106
D6 and pre-trigger	0.856 (0.622–0.948)	6.6 (0.6 to 12.6)	0.0313	0.0939
D2 and pre-trigger	0.703 (0.322–0.901)	2.0 (−3.8 to 7.8)	0.4951	0.4951

Spearman coefficients use 21 women with complete three-visit profiles; CIs are pointwise percentile bootstrap intervals (20,000 resamples of women). Differences are estimates from a linear mixed model of all 80 plasma measurements from 29 women, with visit as a fixed effect and a random intercept for woman. Their CIs are unadjusted Wald intervals; *p* values are shown unadjusted and after Holm adjustment across the three contrasts. An unadjusted CI excluding zero can coexist with an adjusted *p* value above 0.05. The mixed model was added after the complete-case analysis, reported in Supplementary Table S5. ORP, oxidation–reduction potential; CI, confidence interval; D2, cycle day 2; D6, stimulation day 6 (menstrual-cycle day 6 in natural cycles).

**Table 4:** Mature-oocyte counts and embryological proportions among evaluable cycles.

Outcome	Cycles/Women, *n*	Median (IQR)	Range
MII oocytes, count	19/19	6 (4.5–7)	1–13
Oocyte maturation rate, %	19/19	83.3 (76.4–87.5)	57.1–100.0
2PN/recorded-MII proportion, %	16/16	84.5 (69.2–89.7)	50.0–100.0
Recorded blastocyst/2PN proportion, %	17/17	75.0 (42.9–83.3)	0.0–100.0

Proportions were calculated per cycle: MII/retrieved oocytes, 2PN/recorded MII, and day-5 and day-6 blastocysts/2PN. The 2PN/recorded-MII row uses 16 cycles; their confirmed injected counts matched recorded MII. Table S10 separately describes 20 cycles using total injected counts. Extended-culture denominators were unverified, so the blastocyst proportion remains descriptive. Cryopreservation-only cycles contributed only to oocyte outcomes. The cycle with unconfirmed MII was excluded from MII-dependent rows. MII, metaphase II; 2PN, two pronuclei; IQR, interquartile range.

**Table 5:** Associations between follicular-fluid ORP and cycle-level embryological outcomes.

Outcome	Paired Cycles/ Women, *n*	Spearman ρ	95% CI for ρ	BH-Adjusted *p*
Retrieved-oocyte count	25/25	0.230	−0.234 to 0.659	0.4470
Oocyte maturation rate	19/19	−0.383	−0.731 to 0.112	0.3318
2PN/recorded-MII proportion	16/16	−0.393	−0.742 to 0.148	0.3318
Total blastocyst count	17/17	0.119	−0.414 to 0.624	0.6447
Recorded blastocyst/2PN proportion	17/17	−0.215	−0.660 to 0.301	0.5060

Each correlation used one cycle per woman. FF came from one unmatched follicle, whereas outcomes refer to the whole cycle. Associations involving the recorded proportions are descriptive and do not establish laboratory performance or prediction. The 2PN/recorded-MII analysis uses the original 16-cycle subset, not the expanded 20-cycle injected-count cohort. *p* values are two-sided permutation estimates (99,999 permutations), with Benjamini–Hochberg adjustment across five associations. CIs are pointwise percentile bootstrap intervals (20,000 resamples). FF, follicular fluid; ORP, oxidation–reduction potential; BH, Benjamini–Hochberg; CI, confidence interval; MII, metaphase II; 2PN, two pronuclei.

## Data Availability

De-identified data supporting the findings are held by the corresponding author and may be made available upon reasonable request.

## Supplementary Materials

The following supporting information can be downloaded at https://www.mdpi.com/article/10.3390/biomedicines14102404/s1: Table S1: Inventory of analyses: families of estimates, stated aim addressed, multiplicity adjustment, and timing; Table S2: Confidence intervals for median ORP across available observations; Table S3: Completeness, cancellations, and retrieval status; Table S4: Plasma–FF ORP: sensitivity analyses and paired differences; Table S5: Within-cycle plasma ORP: complete-case, sensitivity, and additional mixed-model analyses; Table S6: Third-visit hormones and ORP; Table S7: Post hoc associations of plasma ORP with cycle-level outcomes; Table S8: Sensitivity analyses of the associations between ORP and outcomes; Table S9: Post hoc associations of age with plasma and follicular-fluid ORP; Table S10: Additional clinical descriptors and recorded ICSI counts; Supplementary Methods; separate STROBE reporting checklist.

## Abbreviations

The following abbreviations are used in this manuscript:

2PN	Two pronuclei
AMH	Anti-Müllerian hormone
BH	Benjamini–Hochberg
BMI	Body mass index
CI	Confidence interval
CV	Coefficient of variation
D2	Cycle day 2 sampling visit, before stimulation where applicable
D6	Stimulation day 6 (menstrual-cycle day 6 in natural cycles)
FF	Follicular fluid
ICC	Intraclass correlation coefficient
ICSI	Intracytoplasmic sperm injection
IQR	Interquartile range
IU	International units
IVF	In vitro fertilization
MII	Metaphase II
ORP	Oxidation–reduction potential
PCOS	Polycystic ovary syndrome
SD	Standard deviation
TAC	Total antioxidant capacity
