# Supplementary material for: Exploratory Interim Analysis of Serial Plasma and Single-Follicle Follicular Fluid Oxidation–Reduction Potential During Fertility Treatment
Source: Biomedicines. 2026 Oct 21;14(10):2404. doi: 10.3390/biomedicines14102404 (PMC13702690; doi:10.3390/biomedicines14102404)
Supplement: Supplementary file 1 [file biomedicines-14-02404-s001.zip › 05_STROBE_Checklist.pdf]

STROBE reporting checklist

Relationship Between Serial Plasma and Single-Follicle Follicular Fluid Oxidation–Reduction Potential During Fertility Treatment: A Prospective Observational Pilot Study

Cohort checklist mapped to the revised manuscript and supplement. “Partial” identifies reporting limits that remain explicit in the paper; it does not imply that unavailable records have been recovered. Section and table references are used because pagination may change.

Study design and measurements

Item	Topic	Manuscript location and reporting status
1a	Design named	Title and Abstract. Reported: prospective observational pilot.
1b	Abstract balance	Abstract. Reported: exploratory questions, sample counts, estimates, multiplicity-adjusted results, unquantified assay precision, and cautious conclusions.
2	Clinical context	Section 1. Reported: FF ORP evidence, inconsistent plasma–FF findings and rationale for serial sampling.
3	Questions and hypotheses	Sections 1, 2.1 and 2.6; Table S1. Reported: approved protocol title, aims stated in participant information, and two additional exploratory questions; no confirmatory hypothesis or endpoint hierarchy was prespecified.
4	Design framework	Sections 2.1–2.2. Reported: prospective sampling during treatment, repeated visits and possible repeated cycles.
5	Place and timing	Sections 2.1–2.4. Partial: Fertilia, post-approval sampling, and February–September 2026 dated records reported; calendar dates incomplete for some cycles, and exact visit intervals and processing timestamps unavailable.
6a	Entry and observation	Sections 2.1–2.2 and 3.1; Figure 1 and Table S3. Partial: eligibility criteria and cycle follow-through described; screening and refusal counts unavailable. Pregnancy linkage was not reliable.
6b	Matched groups	Not applicable: no matched exposure groups were constructed.
7	Variable definitions	Sections 2.2 and 2.4–2.5. Partial: ORP, visits and recorded outcomes defined; PCOS/endometriosis ascertainment, dominant-follicle definition and insemination denominators unavailable.
8	Measurement details	Sections 2.2–2.5 and 4.5–4.6; Supplementary Methods. Partial: device and handling described; replicate readings, several processing details and assessor blinding undocumented.
9	Bias safeguards	Sections 2.4–2.6 and 4.2–4.6; Tables S3–S5 and S8. Reported: visible-blood exclusions, woman/cycle rules, missingness checks and remaining measurement and selection bias.
10	Cohort size	Sections 2.1 and 4.7. Partial: all 31 available cycles define the pilot; no power-based design is claimed. Future-study numbers are illustrative only.

STROBE reporting checklist

Analysis and participant accounting

Item	Topic	Manuscript location and reporting status
11	Numeric handling	Section 2.6 and Supplementary Methods. Reported: ORP in mV, count and proportion definitions, ranks, recorded precision and descriptive summaries.
12a	Statistical approach	Section 2.6, Table S1 and Supplementary Methods. Reported: correlations, mixed model, paired tests, resampling and family-specific multiplicity adjustment; no confounder-adjusted prediction model.
12b	Subsets and effect variation	Section 2.6; Tables S4, S5 and S8. Reported: antagonist-only and other sensitivity subsets. No formal interaction analysis was performed.
12c	Incomplete records	Sections 2.5–2.6, 3.1 and 4.4; Figure 1 and Table S3. Reported: analysis-specific denominators, a mixed model of all available plasma measurements and no routine imputation. Poor-response zeros occur only in a stated sensitivity analysis.
12d	Follow-up loss	Sections 2.5 and 4.6; Table S3. Partial: treatment-cycle outcome gaps described. No reliable pregnancy or live-birth analysis; longitudinal follow-up loss cannot be quantified.
12e	Sensitivity checks	Section 2.6 and Supplementary Methods; Tables S3–S5 and S8. Reported: protocol, missing-visit, hemolysis, extreme-value, MII, model-structure and cancellation checks.
13a	Study progression	Section 3.1, Figure 1 and Table S3A. Partial: 29 women, 31 cycles, visits, retrieval and evaluable outcomes reported; eligible and approached counts unavailable.
13b	Non-participation reasons	Section 3.1; Figure 1 and Table S3A–B. Partial: cancellation, untriggered sampling and cryopreservation reasons stated; refusal and some missing-sample or missing-outcome reasons unavailable.
13c	Cohort accounting display	Figure 1 (flow diagram) and Table S3A–B. Reported.
14a	Participant profile	Sections 3.1 and 3.4; Table 1 and Table S6A. Partial: age, BMI, AMH, smoking, protocol and available hormones; indication, antral follicle count and supplement use unavailable.
14b	Availability by variable	Figure 1, Tables 1–5 and S2–S8. Reported: numbers contributing to each analysis, missing smoking, AMH and hormone records, and outcome completeness.
14c	Observation duration	Sections 2.1–2.2 and 4.6. Partial: treatment visits and initial data from the ongoing study stated; patient-level elapsed times and reliable pregnancy follow-up unavailable.

STROBE reporting checklist

Findings and interpretation

Item	Topic	Manuscript location and reporting status
15	Observed outcomes	Sections 3.1 and 3.5; Tables 1, 4 and S3. Reported: oocyte and embryo summaries and cycle completion. Pregnancy and live birth were not analyzed.
16a	Estimates and uncertainty	Sections 3.2–3.5; Tables 2, 3, 5 and S2–S8. Reported: estimates, pointwise intervals and family-specific adjusted p values. Multiplicity adjustment is not confounder adjustment.
16b	Numeric categories	Sections 2.6, 3.1 and 3.4; Tables S3–S5 and S8. Reported: protocol, smoking, completeness and retrieval-status subsets. No ORP clinical cutoff was derived.
16c	Risk translation	Not applicable: the study reports correlations and paired differences, not relative-risk estimates.
17	Additional investigations	Sections 2.6 and 3.2–3.5; Tables S1–S8. Reported: every family of estimates is listed with its timing; sensitivity and post hoc analyses, including stability between visits and cancellation coding, are labeled.
18	Findings against aims	Sections 4.1 and 5. Reported: plasma–FF association, stability of plasma ORP between visits and inconclusive outcome associations.
19	Constraints and bias	Section 4.6. Reported: analytical, selection, precision and denominator limitations. Their magnitude cannot be quantified from the available analytical records.
20	Balanced inference	Sections 4.1–4.7 and 5. Reported: evidence limits, multiplicity, competing explanations and validation needs; no clinical utility claim.
21	Applicability elsewhere	Section 4.6. Reported: small heterogeneous cohort, eligibility limits, exclusion of PCOS and endometriosis, and limited transferability of assay values.
22	Financial support	Funding and Conflicts of Interest statements. Reported: no external funding and no declared conflicts.

Basis and scope

Item numbering follows the STROBE cohort checklist; topic labels are paraphrased. This pilot has continuous ORP exposures rather than matched exposed and unexposed groups. The checklist assesses reporting, not scientific validity or completeness of the underlying records.

Source: STROBE Initiative, cohort checklist. https://www.strobe-statement.org/download/strobe-checklist-cohort-studies-pdf/ (accessed 3 October 2026).
