# Supplementary material for: Exploratory Interim Analysis of Serial Plasma and Single-Follicle Follicular Fluid Oxidation–Reduction Potential During Fertility Treatment
Source: Biomedicines. 2026 Oct 21;14(10):2404. doi: 10.3390/biomedicines14102404 (PMC13702690; doi:10.3390/biomedicines14102404)
Supplement: Supplementary file 1 [file biomedicines-14-02404-s001.zip › biomedicines-4640857-supplementar-resubmit.pdf]

Supplementary Materials

Exploratory Interim Analysis of Serial Plasma and Single-Follicle Follicular Fluid Oxidation–Reduction Potential During Fertility Treatment

These materials report the analysis inventory and the descriptive, sensitivity, and post hoc analyses of the initial available dataset from the ongoing study. Each correlation includes at most one observation per woman. The age analyses use the first available ORP at each stage, selected without regard to value or outcome (Table S9). An unconfirmed MII count is unavailable in main analyses and restored only in Table S8. The original 2PN/recorded-MII correlations use 16 cycles. Additional confirmed AFC and ICSI counts are described in Table S10; the expanded 20-cycle 2PN/total-injected proportion was not subjected to new ORP correlation tests. Extended-culture denominators remain unverified, and outcome proportions are descriptive. No new participants or prospective precision measurements were added in this revision. The Supplementary Methods follow the tables; the STROBE checklist is supplied separately.

Table S1. Inventory of analyses: families of estimates, stated aim addressed, multiplicity adjustment, and timing.

Family of estimates	Estimates, *n*	Stated aim addressed	Multiplicity adjustment	Timing	Reported in
Plasma–FF ORP correlations at the three visits	3	Not a stated aim	Holm	First analysis	Table 2
Plasma–FF sensitivity analyses	8	Not a stated aim	None	First analysis	Table S4
Paired FF minus plasma differences	3	Not a stated aim	None	Added later	Table S4
Plasma–plasma correlations between visits and ICC	4	Not a stated aim	None; no tests	Added later	Table 3; Section 3.3
Mixed-model visit contrasts, all cycles	3	Aim 2	Holm	Added later	Table 3
Mixed-model visit contrasts, antagonist cycles	3	Aim 2	Holm	Added later	Table S5
Mixed-model variant with cycle nested within woman	6	Aim 2	Holm within each model	Added later	Table S5
Complete-case Friedman test and paired visit contrasts	4	Aim 2	Holm across contrasts	First analysis	Table S5
Complete-case sensitivity analyses	11	Aim 2	Holm within each analysis	First analysis	Table S5
FF ORP and embryological outcomes	5	Aim 1	Benjamini–Hochberg	First analysis	Table 5
Plasma ORP and embryological outcomes	15	Aim 1	Benjamini–Hochberg	Added later	Table S7
Plasma ORP changes and profile features with oocyte and blastocyst counts	12	Aim 1	Benjamini–Hochberg	Added later	Table S7

Table S1. Inventory of analyses continued.

Family of estimates	Estimates, *n*	Stated aim addressed	Multiplicity adjustment	Timing	Reported in
Outcome associations with the unconfirmed MII count retained	8	Aim 1	None	Added later	Table S8
Outcome associations in antagonist cycles	8	Aim 1	None	Added later	Table S8
Poor-response cancellations coded as zero retrieved oocytes	2	Aim 1	None; no tests	Added later	Table S3
ORP and smoking, BMI, and AMH	6	Aim 2	None	Added later	Section 3.4
Third-visit estradiol and progesterone with ORP	4	Aim 2	None; no tests	Added later	Table S6
Age and ORP at four sampling stages	4	Aim 2	Holm across four	Expanded during Round 1 revision	Table S9
**Total**	109				

An estimate is a correlation coefficient, a difference with its interval, or a test. No analysis was prespecified; the families and their boundaries were chosen by the authors after data collection. Aim 1, oocyte quality and fertilization in relation to ORP; Aim 2, ORP in relation to the hormonal profile, demographic characteristics, lifestyle, and ovarian stimulation (main text, Section 2.1). First analysis, the initial analysis script; added later, analyses added after the first results were known, during checks and manuscript revision. The FF–outcome family was re-estimated after the unconfirmed MII count was set to missing. Holm adjustment of the mixed-model contrasts and the nested-cycle variant were added in the last revision. Descriptive summaries and CIs for medians (main Tables 1 and 4; Tables S2, S3 and S10) are not counted, and the Fisher z intervals in Table S4 are alternative intervals for the three main correlations. Checks that were computed and not retained are not listed. They include Mann–Whitney comparisons by profile completeness and by retrieval status at cycle level, whose groups were not independent. They also include limits of agreement for the FF–plasma differences and antagonist-only outcome correlations with the unconfirmed MII count retained. ORP, oxidation–reduction potential; FF, follicular fluid; ICC, intraclass correlation coefficient; MII, metaphase II; BMI, body mass index; AMH, anti-Müllerian hormone; CI, confidence interval. During Round 1 revision, the existing two age coefficients were relocated from the participant-characteristics family into an expanded four-stage age family with Holm adjustment (Table S9). Only the D6 and pre-trigger age coefficients are additional estimates. The revised inventory contains 109 estimates in 18 families.

Table S2. Confidence intervals for median ORP across available observations.

Measurement	Cycles, *n*	Women, *n*	Median (IQR), mV	95% CI for median, mV	Range, mV
D2 plasma	31	29	95.6 (81.0–112.0)	89.4–108.0	53.3–145.7
D6 plasma	26	25	94.4 (77.0–115.2)	81.5–109.0	50.4–147.0
Pre-trigger plasma	23	23	103.0 (85.2–113.8)	93.2–107.7	65.9–146.0
Follicular fluid	25	25	115.2 (93.0–128.0)	98.8–124.7	61.8–223.0

CIs are pointwise percentile intervals from 20,000 bootstrap replicates that resampled women as clusters and retained their available cycles. Pre-trigger measurements exclude the untriggered natural-cycle specimen. Means and SDs are given in main Table 1. ORP, oxidation–reduction potential; IQR, interquartile range; CI, confidence interval; D2, cycle day 2; D6, stimulation day 6 (menstrual-cycle day 6 in natural cycles).

Table S3. Completeness, cancellations, and retrieval status.

A. Sampling and outcome completeness

Stage or measurement	Available / denominator	Percent
Enrolled women / recorded treatment cycles	29 women / 31 cycles	—
D2 plasma	31 / 31 cycles	100%
D6 plasma	26 / 31 cycles	83.9%
Pre-trigger plasma	23 / 31 cycles	74.2%
Complete plasma profiles	21 / 31 cycles	67.7%
Oocyte retrieval	25 / 31 cycles	80.6%
FF among retrieval cycles	25 / 25	100%
Confirmed MII count among retrieval cycles	19 / 25	76.0%
Non-cryopreservation retrieval cycles	21 / 25	84.0%
2PN/recorded-MII proportion, non-cryopreservation retrievals	16 / 21	76.2%
Recorded blastocyst outcome, non-cryopreservation retrievals	17 / 21	81.0%

The denominators describe the available cohort; they are not eligibility-adjusted acquisition rates or prespecified feasibility criteria. Some missing samples followed cancellation or the absence of trigger or retrieval. Counts of women screened, approached, or declining, and of rejected aspirates, were unavailable. Four retrieval cycles were for oocyte cryopreservation only. The four other missing blastocyst outcomes had blank embryo records; the reasons could not be established, and they were not imputed as failure. One untriggered natural-cycle third specimen was excluded from the pre-trigger definition.

B. Documented cancellation reasons

Reason recorded in source notes	Canceled cycles, *n*
Poor ovarian response, explicitly stated	3
Low estradiol and low antral follicle count	1
Low estradiol and few follicles	1

Five cancellations occurred in four women; one woman had two canceled cycles before a retrieval cycle. For the zero-oocyte sensitivity analysis, all five documented reasons were classified as poor or low ovarian response. The natural cycle without retrieval was not classified as a poor-response cancellation.

C. ORP by retrieval status, first recorded cycle of each woman

First-cycle status	D2: *n*; median (IQR), mV	D6: *n*; median (IQR), mV
Retrieval	24; 101.5 (89.4–116.6)	22; 100.9 (83.8–117.5)
Poor/low-response cancellation	4; 93.2 (80.0–102.7)	1; 79.9
Natural cycle without retrieval	1; 66.8	1; 63.3

The first recorded cycle was selected for each of the 29 women before data completeness or retrieval status was considered, with no replacement by a later cycle. The woman with three cycles is therefore represented by her first canceled cycle, and her later retrieval is excluded here. This differs from the main retrieval analyses, which include her only retrieval. The single D6 value among canceled cycles does not support a between-group comparison. All summaries are descriptive.

D. Post hoc sensitivity analysis with poor-response cancellations coded as zero retrieved oocytes

Plasma visit	Women, *n*	Spearman ϱ	Pointwise 95% CI
D2	28	0.404	0.019 to 0.685
D6	23	0.424	0.000 to 0.772 *

The first-cycle rule of panel C was retained. The analysis set comprised 24 women who reached retrieval and four with a poor- or low-response cancellation; the natural cycle without retrieval was excluded. Cancellation was coded as zero retrieved oocytes for this analysis only, not as zero fertilization or embryo development. Available plasma values determined n. CIs used 20,000 paired percentile bootstrap resamples. * The D6 lower bound is slightly below zero before rounding, and the interval includes zero. These unadjusted post hoc intervals do not establish an independent or confirmatory association. ORP, oxidation–reduction potential; FF, follicular fluid; MII, metaphase II; 2PN, two pronuclei; IQR, interquartile range; CI, confidence interval; D2, cycle day 2; D6, stimulation day 6 (menstrual-cycle day 6 in natural cycles).

Table S4. Plasma–FF ORP: sensitivity analyses and paired differences.

A. Sensitivity analyses of the associations between plasma and FF ORP

Analysis	Plasma visit	Women, *n*	Spearman ϱ	95% CI for ϱ	Unadjusted *p*
Common cohort with all three plasma measurements and FF	D2	21	0.596	0.115 to 0.877	0.0053
	D6	21	0.758	0.400 to 0.946	0.0001
	Pre-trigger	21	0.848	0.623 to 0.945	<0.0001
Hemolysis-flagged D2 specimen omitted	D2	24	0.533	0.093 to 0.816	0.0080
Antagonist cycles only	D2	20	0.661	0.243 to 0.886	0.0019
	D6	18	0.835	0.532 to 0.960	<0.0001
	Pre-trigger	19	0.900	0.742 to 0.961	<0.0001
Highest FF ORP observation omitted	Pre-trigger	22	0.864	0.651 to 0.952	<0.0001
Main analysis, Fisher z interval	D2	25	0.580	0.208 to 0.806	—
	D6	23	0.715	0.386 to 0.883	—
	Pre-trigger	23	0.866	0.665 to 0.950	—

Each analysis included one cycle per woman. CIs are pointwise percentile bootstrap intervals from 20,000 resamples of paired observations. The Fisher z intervals use the Bonett–Wright standard error (main-text reference [20]), $SE = \sqrt{[(1 + \varrho^2/2)/(n - 3)]}$. p values are two-sided Monte Carlo estimates from 99,999 permutations and are unadjusted.

B. Paired differences between FF and plasma ORP (post hoc)

Plasma visit	Women, *n*	Cycles with FF ORP above plasma ORP	Median difference, mV	95% CI, mV	Unadjusted *p*
D2	25	19 of 25	16.5	5.8 to 33.6	0.0029
D6	23	21 of 23	21.5	11.7 to 32.2	0.0002
Pre-trigger	23	19 of 23	10.4	4.3 to 18.8	0.0002

Differences are FF minus plasma, in mV, with one cycle per woman. CIs for median paired differences use 20,000 paired bootstrap resamples. Unadjusted *p* values are two-sided Wilcoxon sign-permutation estimates. These comparisons across matrices and collection times do not estimate analytical agreement. FF, follicular fluid; ORP, oxidation–reduction potential; CI, confidence interval; D2, cycle day 2; D6, stimulation day 6 (menstrual-cycle day 6 in natural cycles).

Table S5. Within-cycle plasma ORP: complete-case, sensitivity, and additional mixed-model analyses.

**Analysis**	**Contrast**	**Women, *n***	**Difference, mV**	**95% CI, mV**	***p***	**Holm-adjusted *p***
Complete three-visit cohort	D6 − D2	21	−4.0	−13.0 to 4.0	0.1705	0.3409
	Pre-trigger − D2	21	4.5	−2.3 to 12.6	0.5621	0.5621
	Pre-trigger − D6	21	6.7	3.0 to 14.0	0.0050	0.0149
Hemolysis-flagged D2 specimen omitted	D6 − D2	20	−4.5	−13.2 to 4.1	0.1676	0.3352
	Pre-trigger − D2	20	4.2	−5.4 to 11.6	0.6742	0.6742
	Pre-trigger − D6	20	5.6	3.0 to 13.0	0.0074	0.0223
Antagonist cycles only	D6 − D2	17	−4.0	−15.7 to 4.1	0.1630	0.3259
	Pre-trigger − D2	17	4.5	−16.0 to 12.6	0.7467	0.7467
	Pre-trigger − D6	17	4.4	2.9 to 15.3	0.0038	0.0115
All available visit pairs	D6 − D2	25	−3.5	−8.8 to 4.0	0.2357	0.4713
	Pre-trigger − D2	23	4.5	−2.3 to 10.6	0.4867	0.4867
	Pre-trigger − D6	21	6.7	3.0 to 14.0	0.0050	0.0149
Linear mixed model, antagonist cycles	D6 − D2	22 (62 measurements)	−6.2	−13.0 to 0.6	0.0758	0.1769
	Pre-trigger − D2	22 (62 measurements)	0.7	−6.1 to 7.5	0.8399	0.8399
	Pre-trigger − D6	22 (62 measurements)	6.9	−0.3 to 14.0	0.0590	0.1769
Nested mixed model, all cycles	D6 − D2	29 (80 measurements)	−4.0	−9.4 to 1.3	0.1401	0.2802
	Pre-trigger − D2	29 (80 measurements)	2.7	−2.9 to 8.3	0.3482	0.3482
	Pre-trigger − D6	29 (80 measurements)	6.7	0.9 to 12.5	0.0224	0.0672
Nested mixed model, antagonist cycles	D6 − D2	22 (62 measurements)	−5.5	−12.1 to 1.0	0.0994	0.1987
	Pre-trigger − D2	22 (62 measurements)	1.4	−5.1 to 8.0	0.6667	0.6667
	Pre-trigger − D6	22 (62 measurements)	7.0	0.2 to 13.8	0.0451	0.1353

For the paired analyses, the difference is the median paired difference and the CI is a pointwise percentile bootstrap interval from 20,000 replicates. Their p values are from two-sided Wilcoxon signed-rank tests with exact sign permutations, or 99,999 Monte Carlo sign permutations for more than 24 nonzero differences. Holm adjustment covers the three contrasts of each analysis. In the complete cohort, median plasma ORP was 106.0 mV at D2 (IQR, 89.4–116.0), 94.7 mV at D6 (IQR, 83.0–116.9), and 103.7 mV at pre-trigger (IQR, 93.2–116.0). Friedman permutation p values were 0.0045 in the complete cohort (statistic, 10.57; Kendall's W, 0.252), 0.0119 after omission of the flagged specimen, and 0.0067 in antagonist cycles. Rank-biserial correlations for the three complete-cohort contrasts were −0.346, 0.152, and 0.675. In the analysis of all available visit pairs, cycle-specific differences were averaged within each woman. For the linear mixed models, the difference is the model estimate with its Wald 95% CI; visit was a fixed effect, with a random intercept for woman. The nested mixed models added a variance component for cycle nested within woman. The all-cycles model without this component is reported in main Table 3. ORP, oxidation–reduction potential; CI, confidence interval; IQR, interquartile range; D2, cycle day 2; D6, stimulation day 6 (menstrual-cycle day 6 in natural cycles).

Table S6. Third-visit hormones and ORP.

A. Third-visit hormone measurements among retrieval cycles

Variable	Cycles / women, *n*	Median (IQR)	Range
Estradiol, pg/mL	19 / 19	1800.0 (1101.5–2753.0)	136.0–4140.0
Progesterone, ng/mL	19 / 19	0.98 (0.80–1.25)	0.10–6.40

Six retrieval cycles lacked these hormone measurements. Values came from the recorded third-visit fields of the source worksheet. The maximum progesterone value, 6.40 ng/mL, agrees with the cycle-18 worksheet entry. This value was rechecked and confirmed as the clinical laboratory result. The value was retained as recorded. Precise sampling timestamps were unavailable in the analysis records.

B. Post hoc associations of third-visit hormones with ORP

Third-visit hormone	ORP measurement	Women, *n*	Spearman ϱ	Pointwise 95% CI
Estradiol	Pre-trigger plasma	19	−0.104	−0.581 to 0.436
	Follicular fluid	19	−0.279	−0.756 to 0.280
Progesterone	Pre-trigger plasma	19	−0.335	−0.688 to 0.095
	Follicular fluid	19	−0.485	−0.733 to −0.112

All four correlations use the same 19 retrieval cycles from 19 women with both third-visit hormone values, pre-trigger plasma ORP, and FF ORP. Hormone fields corresponded to the recorded trigger visit. Coefficients use average ranks for ties. Pointwise 95% CIs use 20,000 paired percentile bootstrap resamples of women. No *p* values, multiplicity-adjusted intervals, or covariate-adjusted models were computed for this post hoc set. The progesterone–FF interval excludes zero pointwise, but it does not establish a confirmatory or independent association. ORP, oxidation–reduction potential; FF, follicular fluid; IQR, interquartile range; CI, confidence interval.

Table S7. Post hoc associations of plasma ORP with cycle-level outcomes.

A. Plasma ORP at each visit and embryological outcomes

Plasma visit	Outcome	Women, *n*	Spearman ρ	95% CI for ρ	Unadjusted *p*	BH-adjusted *p*
D2	Retrieved-oocyte count	25	0.357	−0.075 to 0.683	0.0811	0.3242
	Oocyte maturation rate	19	0.010	−0.532 to 0.571	0.9688	0.9688
	2PN/recorded-MII proportion	16	−0.375	−0.773 to 0.224	0.1513	0.3242
	Total blastocyst count	17	0.260	−0.235 to 0.659	0.3108	0.5143
	Recorded blastocyst/2PN proportion	17	−0.125	−0.623 to 0.443	0.6306	0.6756
D6	Retrieved-oocyte count	23	0.379	−0.034 to 0.719	0.0749	0.3242
	Oocyte maturation rate	18	−0.385	−0.758 to 0.094	0.1164	0.3242
	2PN/recorded-MII proportion	15	−0.401	−0.783 to 0.168	0.1393	0.3242
	Total blastocyst count	15	0.245	−0.331 to 0.746	0.3771	0.5143
	Recorded blastocyst/2PN proportion	15	−0.183	−0.733 to 0.387	0.5098	0.6373
Pre-trigger	Retrieved-oocyte count	23	0.342	−0.097 to 0.723	0.1098	0.3242
	Oocyte maturation rate	17	−0.240	−0.691 to 0.320	0.3517	0.5143
	2PN/recorded-MII proportion	14	−0.568	−0.858 to 0.000	0.0368	0.3242
	Total blastocyst count	15	0.159	−0.428 to 0.686	0.5678	0.6552
	Recorded blastocyst/2PN proportion	15	−0.333	−0.787 to 0.251	0.2243	0.4205

Each analysis included one cycle per woman. The MII count was unconfirmed in one cycle; its maturation rate and 2PN/recorded-MII proportion were treated as unavailable, and its other observations were retained. Outcome definitions are given in the main text. CIs are pointwise percentile bootstrap intervals from 20,000 paired resamples. *p* values are two-sided Monte Carlo estimates from 99,999 permutations. Benjamini–Hochberg adjustment covered the 15 associations of this panel, separately from the five FF–outcome associations of the main text.

B. Plasma ORP changes and profile features with oocyte and blastocyst counts (Table S7, continued)

Plasma ORP feature	Outcome count	Women, *n*	Spearman ϱ	95% CI for ϱ	Unadjusted *p*	BH-adjusted *p*
D6 − D2	Retrieved oocytes	23	0.184	−0.251 to 0.574	0.4023	0.5205
	Blastocysts	15	−0.198	−0.715 to 0.408	0.4771	0.5205
Pre-trigger − D2	Retrieved oocytes	23	−0.009	−0.455 to 0.459	0.9666	0.9666
	Blastocysts	15	−0.355	−0.784 to 0.248	0.1950	0.4680
Pre-trigger − D6	Retrieved oocytes	21	−0.232	−0.596 to 0.220	0.3114	0.5205
	Blastocysts	13	−0.483	−0.829 to 0.083	0.0970	0.3817
Minimum over three visits	Retrieved oocytes	21	0.386	−0.037 to 0.713	0.0838	0.3817
	Blastocysts	13	0.273	−0.368 to 0.803	0.3698	0.5205
Maximum over three visits	Retrieved oocytes	21	0.489	0.083 to 0.767	0.0256	0.3077
	Blastocysts	13	0.449	−0.163 to 0.846	0.1272	0.3817
Range over three visits	Retrieved oocytes	21	0.172	−0.234 to 0.531	0.4485	0.5205
	Blastocysts	13	0.262	−0.411 to 0.794	0.3910	0.5205

Each correlation included one cycle per woman. The differences and profile features are defined in the Supplementary Methods. Outcomes were the retrieved-oocyte count and the total day-5 and day-6 blastocyst count; missing or non-applicable values were excluded. *p* values and CIs were obtained as in panel A. Benjamini–Hochberg adjustment covered the 12 correlations of this panel as a separate family. Minimum and maximum refer to each woman's measured profile, not to clinical thresholds. ORP, oxidation–reduction potential; MII, metaphase II; 2PN, two pronuclei; BH, Benjamini–Hochberg; CI, confidence interval; D2, cycle day 2; D6, stimulation day 6 (menstrual-cycle day 6 in natural cycles).

Table S8. Sensitivity analyses of the associations between ORP and outcomes.

A. Unconfirmed MII count retained in one cycle

ORP measurement	Outcome	Women, *n*	Spearman ϱ	95% CI for ϱ	Unadjusted *p*
Follicular fluid	Oocyte maturation rate	20	−0.184	−0.656 to 0.335	0.4351
	2PN/recorded-MII proportion	17	−0.480	−0.787 to 0.012	0.0538
D2 plasma	Oocyte maturation rate	20	0.152	−0.407 to 0.657	0.5186
	2PN/recorded-MII proportion	17	−0.474	−0.808 to 0.083	0.0570
D6 plasma	Oocyte maturation rate	19	−0.200	−0.679 to 0.318	0.4101
	2PN/recorded-MII proportion	16	−0.486	−0.811 to 0.024	0.0592
Pre-trigger plasma	Oocyte maturation rate	18	−0.044	−0.595 to 0.502	0.8648
	2PN/recorded-MII proportion	15	−0.639	−0.874 to −0.138	0.0125

The source recorded 10 retrieved oocytes, including 7 germinal-vesicle oocytes, but no explicit MII count. This sensitivity analysis assumes MII = 3 for that cycle and restores its maturation rate and 2PN/recorded-MII proportion; the assumption is not used in the main analysis. The separately confirmed total-injected denominator for this cycle was 10, so its 2PN/total-injected proportion is 3/10; the assumed-MII proportion of 3/3 is a different quantity and is not a verified ICSI rate. Each comparison included one cycle per woman.

B. Antagonist cycles only, oocyte outcomes

ORP measurement	Outcome	Women, *n*	Spearman ϱ	95% CI for ϱ	Unadjusted *p*
Follicular fluid	Retrieved-oocyte count	20	0.276	−0.228 to 0.741	0.2367
	Oocyte maturation rate	15	−0.274	−0.713 to 0.320	0.3208
D2 plasma	Retrieved-oocyte count	20	0.207	−0.273 to 0.565	0.3789
	Oocyte maturation rate	15	0.361	−0.269 to 0.825	0.1834
D6 plasma	Retrieved-oocyte count	18	0.215	−0.268 to 0.656	0.3920
	Oocyte maturation rate	14	−0.121	−0.651 to 0.412	0.6780
Pre-trigger plasma	Retrieved-oocyte count	19	0.293	−0.219 to 0.745	0.2218
	Oocyte maturation rate	14	−0.035	−0.624 to 0.563	0.9069

Analyses were restricted to antagonist cycles, with one cycle per woman and available paired observations. The unconfirmed MII count remained unavailable, as in the main analysis; that cycle still contributed its retrieved-oocyte count. The comparisons in both panels are exploratory sensitivity checks. They use unadjusted two-sided *p* values from 99,999 permutations and pointwise percentile bootstrap CIs from 20,000 paired resamples, and they do not form a confirmatory test family. ORP, oxidation–reduction potential; MII, metaphase II; 2PN, two pronuclei; CI, confidence interval; D2, cycle day 2; D6, stimulation day 6 (menstrual-cycle day 6 in natural cycles).

Table S9. Post hoc associations of age with plasma and follicular-fluid ORP.

ORP measurement	Women n	Spearman ρ	Pointwise 95% CI	Unadjusted p	Holm-adjusted p
D2 plasma ORP	29	0.026	−0.427 to 0.426	0.8944	1.0000
D6 plasma ORP	25	0.110	−0.347 to 0.523	0.6036	1.0000
Pre-trigger plasma ORP	23	0.036	−0.365 to 0.423	0.8711	1.0000
Follicular-fluid ORP	25	0.146	−0.282 to 0.535	0.4840	1.0000

For age associations, the first available ORP at each stage was selected per woman by recorded cycle order before pairwise analysis, without regard to value or outcome. The untriggered natural-cycle third specimen was excluded from pre-trigger analyses. Thus D2 uses the first recorded D2 value, D6 the first available D6 value, and pre-trigger and FF their single available values per woman. Spearman coefficients use average ranks for ties. Pointwise percentile 95% CIs use 20,000 paired bootstrap resamples of women; two-sided p values use 99,999 permutations with a plus-one correction. Holm adjustment covers these four age correlations. The available-case sets differ across stages, so the coefficients do not demonstrate a stage effect. These post hoc estimates and broad intervals do not establish independence from age. ORP, oxidation–reduction potential; FF, follicular fluid; CI, confidence interval.

Table S10. Additional clinical descriptors and recorded ICSI counts.

Measure	Cycles / women, n	Mean ± SD	Median (IQR)	Range
AFC	25 / 25	10.5 ± 4.7	12.0 (10.0–13.0)	1.0–20.0
Total injected oocytes	20 / 20	6.6 ± 3.5	7.0 (4.8–9.3)	1.0–13.0
Recorded 2PN oocytes	20 / 20	5.0 ± 3.0	4.5 (3.0–6.3)	1.0–12.0
2PN/total injected, %	20 / 20	76.1 ± 19.2	81.7 (66.7–86.5)	30.0–100.0

AFC was available for the 25 retrieval cycles from 25 women. Of 21 retrievals not limited to oocyte cryopreservation, 20 had confirmed ICSI and total injected counts; one lacked these fields. Four cryopreservation-only cycles did not contribute to ICSI summaries. Summaries are calculated per cycle, without new correlation tests.

The total-injected denominators are the recorded numbers of oocytes injected, not independently verified MII counts at injection. In the original 16-cycle subset with recorded MII, these denominators matched MII counts and the original 2PN/MII ratios were unchanged. Four additional ICSI records lacked confirmed MII and contribute only to the new descriptive 20-cycle summary. The assumed-MII sensitivity in Table S8 is a separate analysis. SD, standard deviation; IQR, interquartile range; AFC, antral follicle count; ICSI, intracytoplasmic sperm injection; 2PN, two pronuclei.

Supplementary Methods

Procedural Details That Were Not Documented

The main text describes the collection and processing procedure. The available documentation does not establish screening counts or how PCOS/endometriosis were ascertained. The follicle with the largest ultrasound-measured diameter was selected. Individual diameters, aspiration order, flushing or exposure to line medium, anesthetic agent, and oocyte-search conditions were unavailable in the study records. For the filter, only a pore-size range of 1–5 μm was recorded; exact pore size, membrane, and manufacturer were unavailable. Blastocyst grading/usability criteria and whether assessors had access to ORP were undocumented. No objective blood-contamination threshold or assessor-blinding claim is inferred.

The analytic dataset contains recorded sample means, not individual duplicate readings. Residual platelet/cell counts, quantitative hemoglobin, pH, measurement temperature, quantitative quality-control logs, sensor lots, operators, individual air-exposure/measurement timestamps, fasting status, and blood-draw times were unavailable. Samples were measured immediately after air exposure, with a 2-min target, but the window cannot be verified sample by sample without individual timestamps. Additional clinical records documented ICSI and total injected counts in 20 of 21 non-cryopreservation retrievals; one cycle lacked these fields. Extended-culture eligibility and transfers/cryopreservation before day 5 remained unavailable. The hemolysis annotation does not specify its assessment method. Available calendar entries concern menstrual/cycle and retrieval dates, not a complete set of specimen-collection timestamps; some calendar dates are missing, and visit intervals were not reconstructed. The study was not registered, and no fixed final recruitment target was prespecified. An interim stopping rule was not documented in the available study materials. AMH-based eligibility assessment was performed during clinical screening, but numerical results for two women were not transferred to the analysis dataset and remain unavailable for independent rechecking in these records. These are limits of the records used for this report, not evidence that the clinic never recorded the information.

Clinical Descriptors

A qualitative "low AFC" note supported one cancellation classification. The additional clinical records supplied numerical AFC for all 25 retrieval cycles; the six cycles without retrieval had no numerical AFC in these records (Table S10). Total gonadotropin dose entries were present in 23 retrieval cycles. Two formulation-specific entries required unit verification and were excluded, and two natural retrieval cycles had no dose entry. Main Table 1 therefore summarizes recorded totals for the remaining 21 stimulated retrieval cycles, without dose conversion. Available component sums agreed with the recorded totals; administration records were not independently reconciled. Infertility indication, stimulation duration, day of trigger, and supplement exposure remained unavailable for analysis. ICSI counts and AFC are summarized descriptively in Table S10.

Repeated Cycles and Within-Cycle Analyses

One woman contributed three cycles, of which two were canceled before retrieval. Only one of her cycles had FF, evaluable oocyte outcomes, and a complete plasma profile. The complete-profile and ORP–outcome analyses therefore contained one cycle per woman. For the comparison of all available visit pairs, cycle-specific differences were averaged within each woman. Wilcoxon signed-rank tests used exact sign enumeration for no more than 24 nonzero differences and 99,999 Monte Carlo sign permutations otherwise. Holm adjustment covered the three contrasts within each analysis.

The linear mixed models included visit as a fixed effect and a random intercept for woman. They used all available plasma measurements or the antagonist-only subset and were fitted by restricted maximum likelihood. A variant added a variance component for cycle nested within woman. Only one woman contributed more than one cycle, so this component is weakly identified. Wald intervals and p values used the normal distribution. Holm adjustment across the three contrasts of each model was added in the last revision. The missing-data interpretation of these models assumes that data are missing at random, given the model terms. This assumption may not hold when cancellation or missing visits relate to ovarian response. The complete-case analysis uses 21 women and paired rank-based comparisons, whereas the all-available mixed model uses 80 measurements from 29 women and estimates mean visit contrasts. Different populations, estimands, and uncertainty calculations explain why similar point estimates can yield different adjusted p values.

Plasma–FF Comparisons and Participant Characteristics

Paired FF–plasma differences used Wilcoxon signed-rank tests and median paired differences with bootstrap CIs. They compare different matrices collected at different times and do not estimate method agreement. Alternative correlation intervals used the Bonett–Wright Fisher z standard error (main-text reference [20]), $\sqrt{[(1 + \varrho^2/2)/(n - 3)]}$. D2 and FF ORP were related to BMI, AMH, and smoking, using the first recorded D2 measurement per woman and the single retrieval with FF available. Smoking comparisons used unadjusted Mann–Whitney p values. Comparisons by profile completeness were descriptive because one woman contributed cycles to both categories. The retrieval-status comparisons in Table S3 state their separate first-cycle rule. Age analyses are defined below and in Table S9.

Sensitivity Analyses

Sensitivity analyses omitted the hemolysis-flagged D2 measurement, restricted analyses to antagonist cycles, used the common complete-profile cohort, and omitted the highest FF ORP observation. Additional checks restored the unconfirmed MII count. Sensitivity *p* values for correlations were unadjusted, and CIs were pointwise. Pregnancy outcomes were not analyzed, because their linkage to the sampled cycle was uncertain. The source annotation combined low volume and hemolysis without specifying its assessment method; no quantitative hemolysis threshold was inferred.

Between-Visit Plasma ORP Associations

The plasma–plasma correlations in main Table 3 and ICC in Section 3.3 were added post hoc and use 21 women with complete profiles. Spearman coefficients use average ranks for ties. Pointwise percentile 95% CIs use 20,000 resamples of women, retaining all three measurements together. The ICC is ICC(A,1): a two-way mixed-effects model with random women and fixed visits, absolute agreement, and single measurements. Its estimator is (MSwoman − MSerror)/[MSwoman + (k − 1)MSerror + k(MSvisit − MSerror)/n], with n = 21 and k = 3. Mean squares were 1431.957 (woman), 286.110 (visit), and 110.316 (residual). This describes agreement of observed values across visits and combines biological change with analytical variation. It does not measure assay repeatability or establish biological stability. No significance tests were applied to these estimates.

ORP Changes and Profile Features

The analyses in panel B of Table S7 were post hoc and exploratory. Three differences were defined: D6 minus D2, pre-trigger minus D2, and pre-trigger minus D6. They were calculated in mV from all available paired visits. Minimum, maximum, and range (maximum minus minimum) used complete D2, D6, and pre-trigger profiles. The trigger visit varied in timing, so differences were not interpreted as daily slopes. Ratios were not used, because ratios of mV values do not represent proportional changes in oxidative stress. No outcome threshold was chosen, and no receiver-operating-characteristic cutoff or prediction model was developed.

Software and Computation

The first analyses used Python 3.11.15, NumPy 2.4.4, SciPy 1.17.1, and statsmodels for the mixed models; the statsmodels version was not preserved in the saved output. Later corrections and additions used Python 3.12.14 with NumPy 2.3.5 and openpyxl 3.1.5. For the last revision, the mixed models were re-fitted with statsmodels 0.15.0 (Python 3.13.16, NumPy 2.5.3, SciPy 1.18.1) and a tightened convergence tolerance. The estimates agreed with the

earlier output. The reported estimates therefore come from more than one software environment. Derived ORP differences were calculated to the recorded measurement precision (0.1 mV) before rank-based analyses, to preserve ties. Within-woman means of cycle-specific differences retained full precision. Permutations and bootstrap resampling used fixed seeds, with 99,999 permutations and 20,000 bootstrap resamples. Means are arithmetic means, SDs use the sample denominator ($n - 1$), and quartiles use linear interpolation. Means and medians are both reported; a normality test was not used to select between them. The expanded age analysis used Python 3.12.14 and NumPy 2.3.5, with base seed 20261016 and separate deterministic SHA-256-derived streams for each sampling stage and resampling procedure. Its two-sided permutation p values use the plus-one correction; minor differences from previously saved D2/FF age p values reflect the newly specified random streams.

Sample-Size Illustration

For a future study, $n = \text{ceiling}\{3 + (z0.975 + z0.80)^2(1 + \varrho^2/2)/\text{atanh}(\varrho)^2\}$, with the Bonett–Wright variance correction (main-text reference [20]). For two-sided $\alpha = 0.05$ and 80% power, this gives 89 independent women for $\varrho = 0.30$ and 198 for $\varrho = 0.20$. These single-correlation figures do not justify the present cohort retrospectively. They omit missingness, multiplicity, and the requirements of prediction-model development.
